# Knowledge, attitude, and practice of university students towards monkeypox in Bangladesh

**DOI:** 10.1371/journal.pone.0287407

**Published:** 2023-10-12

**Authors:** Md. Akhtarul Islam, Nusrat Jahan Sathi, Sarmistha Paul Setu, Mst. Tanmin Nahar, Md Nafiul Alam Khan, Mahamudul Hasan, Asaduzzaman Khan, Md Mikail Hossen, Md. Mahdi-Al-Muhtasim Nibir, Bayezid Khan, Md. Sabuj Ali, Habib Mohammad Ali, Md. Nazrul Islam, Md. Tanvir Hossain

**Affiliations:** 1 Statistics Discipline, Science Engineering & Technology School, Khulna University, Khulna, Bangladesh; 2 Collaborative Biostatistics Program, School of Public Health, University of Saskatchewan, Saskatoon, Canada; 3 Department of Quantitative Sciences (Statistics), International University of Business Agriculture and Technology, Uttara, Dhaka, Bangladesh; 4 Institute of Mathematical Sciences, Faculty of Science, University of Malaya, Kuala Lumpur, Malaysia; 5 School of Health and Rehabilitation Sciences, Faculty of Health and Behavioural Sciences, School of Health and Rehabilitation Sciences, The University of Queensland, St Lucia, Australia; 6 Mass Communication and Journalism Discipline, Social Science School, Khulna University, Khulna, Bangladesh; 7 Development Studies Discipline, Social Science School, Khulna University, Khulna, Bangladesh; 8 Department of Statistics, Hajee Mohammad Danesh Science &Technology University, Dinajpur, Bangladesh; 9 Department of Media Studies and Journalism, University of Liberal Arts Bangladesh, Dhaka, Bangladesh; 10 Forestry and Wood Technology Discipline, Life Science School, Khulna University, Khulna, Bangladesh; 11 Sociology Discipline, Social Science School, Khulna University, Khulna, Bangladesh; Bangladesh University of Professionals, BANGLADESH

## Abstract

The recent outbreak of viral zoonotic disease–monkeypox–caused by the monkeypox virus, has infected many people worldwide. This study aims to explore the knowledge, attitudes, and practices (KAP) concerning monkeypox among university students in Bangladesh. Data were collected using purposive snowball sampling from 887 university students through an online survey using Google Form. The participants were mostly in their twenties (*M* = 22.33 [SD 2.01] years), and they spent, on average, 2.59 [SD 1.91] hours/day on electronic and social media. The participants generally showed moderate knowledge (39.5%), low attitude (25.1%), and moderate practice (48.6%) toward monkeypox, with 47.6% having had a moderate KAP score. Findings further showed that personal attributes of university students, i.e., age, sex, year of schooling, residence, living status, geographical distribution, e.g., division, were statistically and significantly associated with knowledge, attitudes, and practices regarding monkeypox and overall KAP score. It is also apparent that health status, susceptibility to monkeypox, and exposure to social media were the most common factors significantly associated with knowledge, attitudes, and practices regarding monkeypox and overall KAP score. The current study’s findings underscore the need for developing appropriate information, education, and communication (IEC) materials and their dissemination, which could play an important role in reducing the risk of monkeypox and similar other infectious diseases, particularly among students in Bangladesh.

## Introduction

The coronavirus disease 2019 (COVID-19) is responsible for millions of fatalities worldwide; consequently, it steers panic among people, especially in developing countries like Bangladesh [1–7]. After two years of sustaining the COVID-19 pandemic, the outbreak of another contagious viral disease, monkeypox, has recently reached a worrisome level in several nations [8]. The monkeypox virus causes monkeypox, a severe and rare zoonotic illness belonging to the *Orthopoxvirus* genus of the *Poxviridae* family, and it resembles smallpox [9–11]. The addition of lymphadenopathy symptoms distinguishes it apart from smallpox [9]. Monkeypox was detected for the first time in 1958 in a Danish laboratory among monkeys kept for research purposes [12]. In those colonies of monkeys, two outbreaks of a disease similar to a pox-like illness were observed. Although the origin of the disease is unknown, African rodents and non-human primates (e.g., monkeys) may host the virus and transmit it to humans [12]. In 1970, a 9-month-old infant boy was the first human to be diagnosed with the disease in the Democratic Republic of the Congo [13], and later, it spread to other African nations, especially in Central and West Africa [11], while the first cases outside of Africa being recorded in the United States in 2003 [11, 14]. Israel and the United Kingdom have reported similar figures of confirmed cases of monkeypox in consecutive years, 2018 and 2019 [14, 15]. In May 2019, in Singapore, a Nigerian man was found to have carried the monkeypox virus, the first verified case on the Asian continent [16].

In 2022, according to the World Health Organization (WHO), approximately 1400 monkeypox cases (suspected 1,392 and confirmed 44) were detected in seven African countries [17], relatively higher than the previous two decades [18]. Furthermore, a total of 18,844 cases have been detected in 29 European countries in 2022. In addition, 47 more cases have also been reported from Turkiye and three Western Balkan nations [19]. The WHO reported that the emergence and spread of monkeypox in several non-endemic geographic regions pose a significant threat to public health globally [14, 15]. Recent studies revealed that outbreaks of monkeypox among humans are highly likely, and ‘the public health importance of monkeypox disease should not be underestimated’ [11, 20].

The case-fatality ratio of monkeypox ranges from 0 to 11% in the general population, although it is significantly greater among young children [14]. The death prevalence in recent years has risen due to monkeypox, as reported by the WHO [14]. There are three modes of transmission for monkeypox: (i) animal-to-human; (ii) human-to-human; and (iii) human to substances contained with the monkeypox virus [14, 15, 21]. The virus can spread from infected animals by broken skin (scratches/bites), blood and bodily fluids, or pox lesions [21]. In addition to touching the lesions of an infected person, airborne droplets from coughs and sneezes are also liable for human-to-human transmission [14]. The most prevalent symptoms of monkeypox include fever, headache, muscle aches, backache, swollen lymph nodes, chills, and tiredness with rashes [14]. Recent outbreaks have been characterized by a preponderance of patients with mild clinical manifestations; however, the virus may cause severe disease in young children, pregnant women, and immunosuppressed individuals [14]. In addition, individuals (18 to 50 years of age) are accustomed to suffering uniformly, as public gatherings are relatively common among them [22]. It is essential to pay special attention to university students, as most of this demographic attend educational institutions to acquire knowledge. There is no specific therapy for monkeypox, but antiviral medications and immunizations for smallpox may be helpful, as claimed by some experts [12]. However, the centers and disease control prevention (CDC) prompts several preventative steps to control the spread of monkeypox, particularly in unaffected territories [12]. The most viable preventive measures include isolating infected patients/animals, washing hands after close contact with the infected, and avoiding infected animals and anything contaminated with the virus [12].

Although no case has been identified in Bangladesh, however, the country is surrounded by India, where multiple cases have been identified in recent times. India has established particular instructions to prevent the spread of monkeypox in the states and union territories [23]. Bangladesh has not yet been exposed to vulnerable scenarios generated by monkeypox and has not taken any measures to combat the potential threat posed by monkeypox. However, tension and anxiety about the disease are circulating throughout the country. Understanding knowledge, attitudes, and practices (KAP) related to monkeypox is crucial in societal contexts because it investigates peoples’ norms and behavioral issues regarding the spread of infection. This non-therapeutic approach is an effective way of exploring the thoughts, fears, and perceptions of people regarding a disease [15, 24]. Generally, KAP encompasses many individuals’ beliefs, e.g., its history, knowledge, aggravating factors, symptoms, the availability of therapies, and their effects [20, 25, 26]. These beliefs facilitate the practice of proper prevention-related behavior, which may vary from person to person. Any misperception or incorrect information poses a possible hazard to the nation.

Regarding monkeypox, earlier studies showed that a knowledge gap existed among physicians, health workers, students, and general people [26–30]. To the best of the authors’ knowledge, no studies have explored the KAP of monkeypox in the context of Bangladesh. This understanding is important because mass gatherings promoted the spread of monkeypox, especially among students [22]. Additionally, perception of the real risk changes the behavior of individuals, and it is necessary to monitor the KAP of the students continue to see the changing pattern with changes in the spread level of the diseases [20, 31]. Moreover, to create consciousness among students, it is essential to know the KAP of students regarding monkeypox in order to reduce the spread of this disease. This study aimed to examine KAP regarding monkeypox, whether it is high, moderate or low, among university students in Bangladesh.

## Materials and methods

### Study settings and participants

This cross-sectional study was conducted using Google Form (see S1 File) to assess the Bangladeshi university students’ KAP regarding monkeypox. The students were invited through emails and social media (e.g., Facebook, Messenger, WhatsApp) between 31 May to 22 June, 2022. Inclusion criteria were: (i) a student enrolled in a Bangladeshi university and (ii) having the ability to read the instruction and write the response online using standard English. Considering the aforementioned criteria, the university students were approached, and using the purposive snowball sampling technique each participant was requested to forward the e-questionnaire to friends and others in their peers [11, 20, 32–36]. A total of 887 responses were studied for this study after careful scrutiny.

### Ethical considerations

Ethics for this research was approved by the Khulna University Ethical Clearance Committee (Reference No. KUECC-2022/06/13). The e-questionnaire contained a consent form detailing the rights of the participants, including voluntary participation, anonymity and confidentiality of the participants and their responses, and a right to withdraw without justification within a stipulated timeframe.

### Procedure

The e-questionnaire was developed after an intensive review of relevant literature [12, 14, 17, 24]. The e-questionnaire was modified and adapted from previously published literature on smallpox and monkeypox disease by the World Health Organization (WHO) [14], and from the CDC [12]. The e-questionnaire consisted of five modules: (i) socio-demographic characteristics; (ii) knowledge-related information; (iii) attitude-related information; (iv) practice-related information; and (v) media exposure. Socio-demographic characteristics included age, sex, religion, years of schooling, marital status, living status, residence (rural, sub-urban and urban), and division, while the KAP modules contained 26 five-point Likert scale items regarding knowledge (10 items), attitude (8 items) and practice (8 items) toward monkeypox. For knowledge and attitude-related items, possible responses ranged from ‘strongly disagree’ to ‘strongly agree’, whereas, for practice items, responses were ‘never’ to ‘always with an additional response of ‘not applicable’. The fifth module included items on media exposure, such as how frequently the participants received news from the radio, television, Facebook, WhatsApp, Twitter, and other sources regarding monkeypox.

### Score generation (KAP)

The e-questionnaire was divided into five interrelated but distinct modules: module 1 contained the socioeconomic background and health status of the participants (e.g., age, sexual identity, religion, year of schooling, residence, division, marital status, living status, health status, time spent (hour) on media, and ever thought that the participants had Monkeypox cases or ever felt affected by monkeypox); module 2, module 3 and module 4 included questions on knowledge (10 items), attitude (8 items) and practice (8 items), respectively. The knowledge information concerning monkeypox disease had questions about signs, transmission (e.g., Human-to-human transmission occurs only through large respiratory droplets), duration of infection (e.g., The illness typically lasts for 2–4 weeks), people at risk (e.g., Homosexuals, bisexuals, and people with multiple sex partners are more at risk). The attitude module emphasized isolation from infected persons, avoiding clothing or bedding of someone with the rash, and measures against the infection. The practice module had questions mainly on avoiding infected persons or sick animals’ practices maintained by the participants. For analysis, the responses of the 26 items were subsequently recoded as 1 for accurate/correct responses and 0 for inaccurate/incorrect responses (including neutral responses) (see Table 2). Later, the score for each category was re-categorized into three levels, namely low, moderate, and high score. Finally, quartiles were used to classify the total score: up to the 25^th^ quartile was a low score, 26^th^ to 75^th^ quartile was a moderate score and more than the 76^th^ quartile was a high score in every module (knowledge, attitude, and practice) [32].

### Statistical analyses

The data were analyzed using IBM SPSS Statistics v27. Initially, descriptive statistics, such as frequency, percentage, mean, and standard deviation (SD), of socio-demographic aspects, were used to elaborate on the basic characteristics of the participants. In order to analyze the association between socio-demographic features and scores on knowledge, attitude, and practice regarding monkeypox, Pearson’s Chi-square test of independence (χ^2^) was utilized. The dependent variable has three categories, namely low, medium, and high scores. So finally, the generalized ordered logistic regression model [37] was implemented to estimate the score of knowledge, attitude, practice, and total KAP score of monkeypox among university students in Bangladesh. For all statistical analyses, the probability value of α level (significance) was 0.05.

## Results

### Socio-demographic features

Table 1 shows the socio-demographic characteristics of the participants. There was more male (535) than female (352) participants; a total of 2.6% (self-reported percentage) were found to have ever thought that they had monkeypox cases. The average age of the participants was 22.33, and a total of 571 participants were living in urban areas, mostly with their families (424). The majority of students were from the Khulna division (449), while others (49.4) resided in other divisions in Bangladesh. Again, most of them (807) received up to a Bachelor degree of schooling. About 566 of the study participants spent ≤2 hours/day on electronic and social media. Table 1 summarizes the remaining socio-demographic details of the participants.

**Table 1:** Socio-demographic features.

Variables	n (%)	*M* & SD
Age (in Year)		
≤20	144 (16.2)	22.33 & 2.01
21–23	515 (58.1)	
≥24	228 (25.7)	
Sex identity		
Male	535 (60.3)	
Female	352 (39.7)	
Religion		
Islam	744 (83.9)	
Hindu	125 (14.1)	
Others	18 (2.0)	
Years of schooling		
Honor’s	807 (91.0)	
Master’s ≥	80 (9.0)	
Residence		
Rural	141 (15.9)	
Sub-urban	175 (19.7)	
Urban	571 (64.4)	
Living status		
Alone	186 (21.0)	
With friends	277 (31.2)	
With family	424 (47.8)	
Division		
Khulna	449 (50.6)	
Others	438 (49.4)	
Marital status		
Never married	800 (90.2)	
Ever married	87 (9.8)	
Health status		
Poor	115 (13.0)	
Good	473 (53.3)	
Excellent	299 (33.7)	
Time spent (hour) on media		
≤ 2 hours	566 (63.8)	2.59 & 1.91
3–4 hours	173 (19.5)	
5 hours and above	148 (16.7)	
Ever felt affected by monkeypox		
No	816 (92.0)	
Yes	23 (2.6)	
Maybe / not sure	48 (5.4)	

**Note**. ^***M***.^ Mean; ^**SD**.^ Standard deviation

### Evidence on participants’ knowledge scores, attitude scores, and practice scores regarding monkeypox

Table 2 shows the association of different socio-demographic factors with knowledge, attitudes, and practices score classifications (low, moderate, high) regarding monkeypox. The majority of individuals had a moderate (39.5%) knowledge score, followed by a high score (24.1%). Many participants’ knowledge levels on monkeypox were shown to be significantly related to their age, years of schooling, living status, marital status, health status, and time spent (hour) on media. Suggestively, the higher number of the 21–23 age group were counted in high (13.5%) and moderate (24.0%) knowledge scores classification compared to their equivalent ≤20 age group participants in high (5.5%) and moderate (5.3%), respectively. Honor’s students had a considerably higher knowledge score of monkeypox (22.9%). It is also evident that participants living with families showed relatively higher knowledge (high 12.7% and moderate 18.0% knowledge score). A significant number of participants (38.1%) had a moderate attitude score, followed by high scores (36.8%). Many participants’ attitude levels toward monkeypox were shown to be significantly related to their sexual identity, residence, living status, time spent (hour) on media, and ever felt affected by monkeypox. Suggestively, a higher number of females were counted in high (18.6%) and moderate (12.4%) attitude scores classification compared to their equivalent males in high (18.2%) and moderate (25.7%), respectively. Urban resided students showed significant moderate attitude score (24.9%) towards monkeypox. In the case of living with family, students showed better attitudes towards monkeypox (high 16.2% and moderate 20.1% attitude score). Again, many participating students exhibited low practice scores (26.5%), whereas 48.6% of the participants showed moderate practice scores. Most of the participants’ practice levels on monkeypox were shown to be significantly related to their religion, time spent (hour) on media, and ever felt affected by monkeypox. A substantial practice score was identified among Muslims (high 19.5% and moderate 41.4% practice score). The perceived high (14.7%) practice towards monkeypox was in the participants who spent ≤ 2 hours daily to get information on media.

**Table 2:** Associations between socio-demographic aspects and monkeypox knowledge score, attitude score, practice score and total KAP score attitude score categories.

Variables	Knowledge Score N (%)			*P*-value	Attitude Score N (%)			*P*-value	Practice Score N (%)			*P*-value	KAP Score N (%)			*P*-value
	Low	Moderate	High		Low	Moderate	High		Low	Moderate	High		Low	Moderate	High	
Total	323 (36.4)	350 (39.5)	214 (24.1)		223 (25.1)	338 (38.1)	326 (36.8)		235 (26.5)	431 (48.6)	221 (24.9)		246 (27.7)	422 (47.6)	219 (24.7)	
Age (in Year)																
≤20	48 (5.4)	47 (5.3)	49 (5.5)	.020	38 (4.3)	43 (4.8)	63 (7.1)	.217	34 (3.8)	67 (7.6)	43 (4.8)	.566	40 (4.5)	58 (6.5)	46 (5.2)	.184
21–23	182 (20.5)	213 (24.0)	120 (13.5)		130 (14.7)	206 (23.2)	179 (20.2)		143 (16.1)	248 (28.0)	124 (14.0)		140 (15.8)	251 (28.1)	124 (14.0)	
≥24	93 (10.5)	90 (10.1)	45 (5.1)		55 (6.2)	89 (10.0)	84 (9.5)		58 (6.5)	116 (13.1)	54 (6.1)		66 (7.4)	113 (12.7)	49 (5.5)	
Sex identity																
Male	198 (22.3)	211 (23.8)	126 (14.2)	.854	146 (16.5)	228 (25.7)	161 (18.2)	.000	147 (16.6)	244 (27.5)	144 (16.2)	.077	160 (18.0)	255 (28.7)	120 (13.5)	.079
Female	125 (14.1)	139 (15.7)	88 (9.9)		77 (8.7)	110 (12.4)	165 (18.6)		88 (9.9)	187 (21.1)	77 (8.7)		86 (9.7)	167 (18.8)	99 (11.2)	
Religion																
Islam	276 (31.1)	285 (32.1)	183 (20.6)	.577	188 (21.2)	285 (32.1)	271 (30.6)	.832	204 (23.0)	367 (41.4)	173 (19.5)	.005	209 (23.6)	358 (40.4)	177 (20.0)	.269
Hindu	41 (4.6)	56 (6.3)	28 (3.2)		31 (3.5)	48 (5.4)	46 (5.2)		26 (2.9)	61 (6.9)	38 (4.3)		32 (3.6)	59 (6.7)	34 (3.8)	
Others	6 (0.7)	9 (1.0)	3 (0.3)		4 (0.5)	5 (0.6)	9 (1.0)		5 (0.6)	3 (0.3)	10 (1.1)		5 (0.6)	5 (0.6)	8 (0.9)	
Years of schooling																
Honor’s	385 (32.1)	319 (36.0)	203 (22.9)	.032	199 (22.4)	309 (34.8)	299 (33.7)	.571	212 (23.9)	389 (43.9)	206 (23.2)	.409	217 (24.7)	388 (43.7)	202 (22.8)	.202
Master’s ≥	38 (4.3)	31 (3.5)	11 (1.2)		24 (2.7)	29 (3.3)	27 (3.0)		23 (2.6)	42 (4.7)	15 (1.7)		29 (3.3)	34 (3.8)	17 (1.9)	
Residence																
Rural	60 (6.8)	46 (5.2)	35 (3.9)	.262	49 (5.5)	43 (4.8)	49 (5.5)	.047	41 (4.6)	57 (6.4)	43 (4.8)	.295	47 (5.3)	64 (7.2)	30 (3.4)	.202
Sub-urban	58 (6.5)	69 (7.8)	48 (5.4)		38 (4.3)	74 (8.3)	63 (7.1)		44 (5.0)	88 (9.9)	43 (4.8)		40 (4.5)	94 (10.6)	41 (4.6)	
Urban	205 (23.1)	235 (26.5)	131 (14.8)		136 (15.3)	221 (24.9)	214 (24.2)		150 (16.9)	286 (32.2)	135 (15.2)		159 (17.9)	264 (29.8)	148 (16.7)	
Living status																
Alone	77 (8.7)	80 (9.0)	29 (3.3)	.045	56 (6.3)	71 (8.0)	59 (6.7)	.010	58 (6.5)	85 (9.6)	43 (4.8)	.407	60 (6.8)	92 (10.4)	34 (3.8)	.206
With friends	95 (10.7)	110 (12.4)	72 (8.1)		65 (7.3)	123 (13.9)	89 (10.0)		65 (7.3)	144 (16.2)	68 (7.7)		75 (8.5)	130 (14.7)	72 (8.1)	
With family	151 (17.0)	160 (18.0)	113 (12.7)		102 (11.5)	144 (16.2)	178 (20.1)		112 (12.6)	202 (22.8)	110 (12.4)		111 (12.5)	200 (22.5)	113 (12.7)	
Division																
Khulna	169 (19.1)	175 (19.7)	105 (11.8)	.728	119 (13.4)	176 (19.8)	154 (17.4)	.294	113 (12.7)	212 (23.9)	124 (14.0)	.164	128 (14.4)	206 (23.2)	115 (13.0)	.589
Others	154 (17.4)	175 (19.7)	109 (12.3)		104 (11.7)	162 (18.3)	172 (19.4)		122 (13.8)	219 (24.7)	97 (10.9)		118 (13.3)	216 (24.4)	104 (11.7)	
Marital status																
Never married	288 (32.5)	327 (36.9)	185 (20.9)	.019	194 (21.9)	312 (35.2)	294 (33.1)	.117	207 (23.3)	398 (44.9)	195 (22.0)	.111	218 (24.6)	386 (43.5)	196 (22.1)	.452
Ever married	35 (3.9)	23 (2.6)	29 (3.3)		29 (3.3)	26 (2.9)	32 (3.6)		28 (3.2)	33 (3.7)	26 (2.9)		28 (3.2)	36 (4.1)	23 (2.6)	
Health status																
Poor	50 (5.6)	44 (5.0)	21 (2.4)	.042	29 (3.3)	33 (3.7)	53 (6.0)	.063	30 (3.4)	61 (6.9)	24 (2.7)	.629	30 (3.4)	58 (6.5)	27 (3.0)	.538
Good	165 (18.6)	176 (19.8)	132 (14.9)		115 (13.0)	180 (20.3)	178 (20.1)		124 (14.0)	222 (25.0)	127 (14.3)		127 (14.3)	219 (24.7)	127 (14.3)	
Excellent	108 (12.2)	130 (14.7)	61 (6.9)		79 (8.9)	125 (14.1)	95 (10.7)		81 (9.1)	148 (16.7)	70 (7.9)		89 (10.0)	145 (16.3)	65 (7.3)	
Time spent (hour) on media																
≤ 2hr	182 (20.5)	237 (26.7)	147 (16.6)	.008	111 (12.5)	233 (26.3)	222 (25.0)	< .001	127 (14.3)	309 (34.8)	130 (14.7)	< .001	128 (14.4)	287 (32.4)	151 (17.0)	< .001
3-4hrs	74 (8.3)	66 (7.4)	33 (3.7)		51 (5.7)	64 (7.2)	58 (6.5)		50 (5.6)	72 (8.1)	51 (5.7)		53 (6.0)	86 (9.7)	34 (3.8)	
5hrs and above	67 (7.6)	47 (5.3)	34 (3.8)		61 (6.9)	41 (4.6)	46 (5.2)		58 (6.5)	50 (5.6)	40 (4.5)		65 (7.3)	49 (5.5)	34(3.8)	
Ever felt affected by monkeypox																
No	294 (33.1)	322 (36.3)	200 (22.5)	,813	191 (21.5)	318 (35.9)	307 (34.6)	.004	191 (21.5)	318 (35.9)	307 (34.6)	.001	214 (24.1)	397 (44.8)	205 (23.1)	.018
Yes	8 (0.9)	10 (1.1)	5 (0.6)		13 (1.5)	6 (0.7)	4 (0.5)		13 (1.5)	6 (0.7)	4 (0.5)		11 (1.2)	7 (0.8)	5 (0.6)	
Maybe / not sure	21 (2.4)	18 (2.0)	9 (1.0)		19 (2.1)	14 (1.6)	15 (1.7)		19 (2.1)	14 (1.6)	15 (1.7)		21 (2.4)	18 (2.0)	9 (1.0)	

Table 2 also indicates the KAP score classifications (low, moderate, high) regarding monkeypox. A significant proportion of participants (47.6%) had a moderate KAP score, followed by high scores (24.7%). The majority of the participants’ monkeypox KAP scores were shown to be substantially associated with time spent (hour) on media and ever felt affected by monkeypox. The perceived high (17.0%) KAP towards monkeypox was among people who spent 2 hours or less per day obtaining information from the media regarding monkeypox. It was also observed significantly in the moderate (44.8%) KAP score of the participants who had ever thought they had monkeypox symptoms or had monkeypox-related effects.

Table 3 indicates that the generalized ordered logit (GOL) analysis of the KAP level of the students concerning monkeypox with an adjusted odds ratio (aOR) with a 95% confidence level. A significant GOL regression was found for knowledge levels (LR χ^2^ = 72.85), attitude levels (LR χ^2^ = 102.04), practice levels (LR χ^2^ = 86.28), and overall KAP levels (LR χ2 = 72.01) at 1% level of significance. In this model, age, years of schooling, residence, living status, marital status, health status, and time spent (hour) on media were significantly associated with students’ knowledge levels about monkeypox. When compared to the age group less than or equal to 20 years, the odds for the 21–23 year age group were (aOR: 0.53, 95% CI = .35, .80) less likely of being in the high knowledge group of monkeypox than the low or moderate knowledge group. Again, compared to students from rural residences, there were 1.55 times (aOR: 1.55, 95% CI = .96, 2.49) more likely to be in the moderate and high knowledge group of monkeypox for students from sub-urban residences than the low knowledge group. In the case of comparing the high knowledge group to the low or moderate knowledge group, the ever-married students were 1.97 times (aOR: 1.97, 95% CI = 1.19, 3.26) more likely to be in the high knowledge group of monkeypox than never-married students. Again, comparing the students with poor health status, the observed knowledge for the students with good health was 1.65 times more (aOR: 1.65, 95% CI = .98, 2.80) likely to be in the high knowledge group than in the low or moderate knowledge group.

**Table 3:** Generalized ordered logit estimates of knowledge, attitude, and practice (KAP) level with factors associations concerning monkeypox.

Variables	Knowledge		Attitude		Practice		KAP score	
	Low vs Moderate & High aOR [95%CI]	High vs Low or Moderate aOR [95%CI]	Low vs Moderate & High aOR [95%CI]	High vs Low or Moderate aOR [95%CI]	Low vs Moderate & High aOR [95%CI]	High vs Low or Moderate aOR [95%CI]	Low vs Moderate & High aOR [95%CI]	High vs Low or Moderate aOR [95%CI]
**Age (in Year)**								
≤20 ^**r**^								
21–23	.92[.62, 1.38]	.53[.35, .80] *	1.26[.81, 1.96] *	.78[.53, 1.15]	.96[.61, 1.51]	.72[.47, 1.11]	1.13[.74, 1.74]	.69[.46, 1.05]
≥24	.79[.48, 1.30]	.48[.28, .82] *	1.42[.82, 2.46]	.95[.58, 1.55]	1.18[.68, 2.05]	.84[.49, 1.43]	1.21[.71, 2.04]	.61[.36, 1.05] *
**Sex identity**								
Male ^**r**^								
Female	1.01[.74, 1.38]	.85[.60, 1.21]	1.36[.96, 1.94]	1.79[1.32, 2.44] *	1.14[.81, 1.60]	.69[.48, .98] *	1.28[.91, 1.79]	1.20[.85, 1.69]
**Religion**								
Islam ^**r**^								
Hindu	1.32[.87, 2.00]	.85[.53, 1.36]	1.22[.77, 1.94]	1.15[.76, 1.73]	1.37[.85, 2.22]	1.34[.87, 2.07]	1.26[.80, 1.99]	1.21[.78, 1.89]
Others	1.12[.41, 3.09]	.61[.17, 2.20]	.91[.29, 2.91]	1.90[.72, 4.98]	.72[.24, 2.14]	5.00[1.88, 13.33] *	1.01[.34, 2.98]	2.60[.98, 6.93] *
**Years of schooling**								
Honor’s ^**r**^								
Master’s ≥	.60[.35, 1.03] *	.52[.25, 1.08]	.70[.39, 1.26]	.77[.44, 1.33]	.78[.44, 1.40]	.61[.32, 1.19]	.56[.32, .99] *	.91[.48, 1.74]
**Residence**								
Rural ^**r**^								
Sub-urban	1.55[.96, 2.49] *	1.26[.74, 2.13]	1.83[1.08, 3.10] *	1.10[.68, 1.79]	1.13[.67, 1.89]	.90[.53, 1.51]	1.63[.96, 2.75] *	1.25[.72, 2.19]
Urban	1.40[.94, 2.07]	.98[.62, 1.55]	1.59[1.05, 2.41] *	1.12[.75, 1.68]	1.00[.65, 1.54]	.80[.52, 1.23]	1.22[.80, 1.85]	1.30[.81, 2.08]
**Living status**								
Alone ^**r**^								
With friends	1.29[.87, 1.91]	1.87[1.15, 3.06] *	1.47[.95, 2.27]	1.11[.73, 1.68]	1.62[1.05, 2.50] *	.92[.58, 1.45]	1.35[.88, 2.06]	1.64[1.02, 2.64] *
With family	1.26[.87, 1.83]	2.02[1.26, 3.25] *	1.41[.94, 2.13]	1.43[.97, 2.10] *	1.44[.96, 2.15]	1.23[.80, 1.89]	1.39[.93, 2.09]	1.66[1.05, 2.62] *
**Division**								
Khulna ^**r**^								
Others	1.27[.94, 1.72]	1.03[.73, 1.45]	1.21[.87, 1.68]	1.20[.89, 1.62]	.87[.63, 1.21]	.70[.50, .98] *	1.11[.81, 1.54]	.94[.67, 1.31]
**Marital status**								
Never married ^**r**^								
Ever married	.95[.59, 1.52]	1.97[1.19, 3.26] *	.62[.37, 1.05]	.90[.55, 1.47]	.88[.53, 1.47]	1.56[.93, 2.63]	.84[.50, 1.39]	1.22[.71, 2.09]
**Health status**								
Poor ^**r**^								
Good	1.37[.89, 2.10]	1.65[.98, 2.80] *	.90[.55, 1.48]	.65[.42, 1.00] *	.95[.59, 1.54]	1.39[.83, 2.33]	.92[.57, 1.49]	1.04[.63, 1.69]
Excellent	1.30[.82, 2.06]	1.11[.63, 1.96]	.83[.49, 1.41]	.57[.36, .90] *	.90[.54, 1.51]	1.19[.69, 2.07]	.84[.50, 1.40]	.84[.49, 1.42]
**Time spent (hour) on media**								
≤ 2hr ^**r**^								
3-4hrs	.60[.42, .86] *	.64[.41, .99] *	.57[.38, .85] *	.83[.57, 1.20]	.66[.44, .98] *	1.51[1.02, 2.25] *	.66[.44, .97] *	.69[.45, 1.05]
5hrs and above	.54[.37, .79] *	.82[.53, 1.28]	.37[.25, .55] *	.72[.48, 1.07]	.48[.32, .73] *	1.15[.74, 1.78]	.39[.26, .57] *	.81[.52, 1.27]
**Ever felt affected by monkeypox**								
No ^**r**^								
Yes	1.50[.61, 3.68]	1.20[.42, 3.38]	.36[.15, .88] *	.44[.15, 1.35]	.69[.29, 1.65]	1.66[.68, 4.07]	.63[.26, 1.50]	1.08[.38, 3.05]
Maybe / not sure	.72[.39, 1.32]	.69[.32, 1.50]	.41[.22, .77] *	.71[.37, 1.36]	.48[.25, .89] *	1.20[.60, 2.38]	.41[.22, .77] *	.67[.31, 1.43]
**LR χ2**	72.85 **		102.04**		86.28**		72.01**	

Note. ^**Bold and star**.^ Values indicate the probability value <0.05; ^**Double star**.^ Values indicate the probability value <0.001; ^**aOR**.^ Adjusted odds ratio; ^**r**.^ Reference group; ^**LR**.^ Likelihood Ratio; ^**χ2**.^ Chi-square

Again, in this model, age, sex identity, residence, living status, health status, time spent (hour) on media, and ever felt affected by monkeypox were found to be significantly associated with attitude levels among students towards monkeypox. When compared to the low altitude group students, there exist 1.26 times (aOR: 2.21, 95% CI = 1.15, 4.04) more likely moderate or high attitude in the 21–23 age group students about monkeypox than those in the ≤20 age group. Female students were 1.79 times (aOR: 1.32, 95% CI = 1.30, 1.91) more likely to have a low or moderate attitude level than male students who had high attitude levels. Concerning monkeypox, the odds of attitude level for sub-urban students were 1.83 times more likely (aOR: 1.83, 95% CI = 1.08, 3.10) to be in the moderate or high group than the low attitude group for rural students.

It is also apparent in the model that sex identity, religion, living status, division, time spent (hour) on media, and ever felt affected by monkeypox were found to be significantly associated with practice levels among students towards monkeypox. It demonstrates that female students were (aOR: 0.69, 95% CI = .48, .98) less likely to be in the high practice group when compared to those in the low or moderate practice group concerning monkeypox than male students. Other religious students were five times more likely (aOR: 5.00, 95% CI = 1.88, 13.33) to be in the low or moderate group than the high practice group for Islam religious students.

Moreover, age, religion, years of schooling, residence, living status, time spent (hour) on media, and ever felt affected by monkeypox were significantly associated with overall KAP levels among students towards monkeypox. In comparison to the age group less than or equal to 20 years, the odds for the≥24 years age group was (aOR: .61, 95% CI = .36, 1.05) less likely to be in the high knowledge group of monkeypox than low or moderate KAP scores group. In the case of moderate or high KAP scores against low KAP levels, master’s and above level of education were (aOR: 0.56, 95% CI = .32, .99) less likely to be in low KAP level group towards monkeypox compared to Honor’s. It demonstrates that individuals who lived with their families were 1.66 times (aOR: 1.66, 95% CI = 1.05, 2.62) more likely to be in the high KAP level group than those in the low or moderate KAP concerning monkeypox than those who lived alone. The odds of KAP levels for people who spent 3–4 hours per day getting information or getting updated from the media about monkeypox were less (aOR: .66, 95% CI = .44, .97) likely to be in the low than moderate or high KAP level group compared to those who spent ≤ 2 hours per day on media.

## Discussion

After recovering from the COVID-19 pandemic, the recent outbreak of monkeypox poses a significant threat to the overall wellbeing of people worldwide. This study, therefore, was designed to measure the knowledge, attitude, and practice (KAP) of Bangladeshi university students towards monkeypox and to identify the key factors that determine the levels of KAP among them regarding monkeypox. Evidence suggests that proper knowledge, positive attitude, and better practice among students regarding an outbreak can ensure a healthy and safe life with reduced stress [38].

Although the results of this study revealed that about 63.6% of students had relatively higher knowledge about monkeypox; previous study results showed that the perception of monkeypox was not satisfactory among most university students [27, 39, 40]. The unprecedented loss of life, particularly the relatives, during COVID-19, might have encouraged university students to know more about a similar contagious outbreak, such as monkeypox, to take protective and preventive measures for themselves and their loved ones. Our study also revealed that age, residence, living status, marital status, and health status had a significant positive association with the knowledge score of students regarding monkeypox. This finding is parallel with the previous studies [33, 41]. The students who are at a younger age are more likely to have higher knowledge compared to the older students. This may be because older students generally think more about their careers than younger students and concentrate less on other subjects [42, 43]. Besides, unlike younger university students, older students were less likely to get information from online sources [44]. The students who lived in sub-urban and urban locations were more likely to have moderate to high knowledge of the risk of monkeypox than those from rural areas [45]. The reason behind this is that during any pandemic outbreak, the local authorities and government take necessary steps and strategies primarily for urban areas where the epidemic frequently spreads quickly. As a result, rural areas become the victim of deprivation of the required information and preventive or protective strategies [46]. The students with relatively good health had higher knowledge of monkeypox than those with poor health conditions. One of the prior studies also suggested that during the pandemic, health consciousness increased [47], and for this reason, the students with good health conditions acquire more knowledge regarding the potential risk factors and behaviors associated with infectious diseases. This study also suggested that the ever-married students had higher knowledge regarding monkeypox than never-married students, which is analogous to an earlier study [48].

The findings also showed that three out of four university students (74.9%) had a higher level of attitude about the risk factors associated with monkeypox. This finding supports the previous studies, where students had a greater positive attitude toward Dengue fever [49] and COVID-19 [38]. This study also disclosed a significant positive association of participants’ age, sex, residence, and living status with the attitude score of students regarding monkeypox. But participants’ health status and time spent on media had a significant negative relation with the attitude score of students regarding the risk of monkeypox. Some earlier studies also identified that age, sex, residence, and living status were significantly associated with the attitude score regarding the pandemic [41, 50–52]. This study suggested that students who lived with their family members had a higher (36.3%) attitude toward the risk of monkeypox. An earlier study suggested that the shared support gained from one’s family member significantly and positively impacted the attitude regarding social distancing [50]. In this study, male participants had a greater attitude (43.9%) regarding monkeypox than their female counterparts (31%), which is similar to the results of another study [53], suggesting a more positive attitude toward COVID-19 among males than females. This result is inconsistent with an earlier study that indicated that female participants had a better attitude than male participants regarding dengue fever [49].

In the case of practice regarding monkeypox, most of the students (73.5%) strictly maintained necessary preventive measures to protect themselves from the risk of possible monkeypox infection (i.e., most of the students practiced higher [high to moderate] preventive measures). The protective measures include avoiding contact with sick or dead animals with symptoms, or anyone who has a rash that includes blisters or pus-filled patches, washing hands frequently with soap and water or using an alcohol-based hand sanitizer, eating thoroughly cooked meat, wearing a face mask, maintain safe sex, avoid sneezing in public spaces, and avoid sharing cigarettes, as such were advocated by health agencies to minimize the risk of monkeypox [54, 55]. In addition, the results of our study indicate that students’ sex, religion, living status, division, and spending time on media exhibited a significant association with the practice score regarding monkeypox. Female students had better practice regarding monkeypox than their male counterparts. Another study also found that female participants practiced better than male participants [53, 56].

In the case of overall KAP scores, students living in the urban area and staying with friends and family significantly improved overall KAP scores (knowledge, attitude, and practices) about monkeypox. Male students had a higher score (13.5%) than female students (11.2%), and the students who lived with their families had a higher KAP score than those who lived alone.

### Strengths and limitations

This study has certain limitations. Using the purposive snowball sampling technique, a nonrandom and nonprobability approach to select participants, may not allow the generalization of the findings. The participants who responded to the e-questionnaire were instructed to forward it to their friends and other students in their contacts, so the recruitment bias may also have happened. Most participants were from the Khulna division, so the results may not appropriately be epitomized for the entire population of Bangladesh. Moreover, as this study was conducted among educated participants with access to the Internet, the results may only represent the educated youths of Bangladesh, especially university students. Therefore, the self-reported responses may not be similar to that of the entire populace. Since this study was cross-sectional in nature, the results described represent the situation of a particular period, i.e., the survey period. The KAP among university students is also subject to change with the changing situation of monkeypox. Thus, a longitudinal study with a nationally representative sample is required to examine the time-dependent KAP changes and the associated risk factors, which may give a more extended area of research on this topic. Considering this population’s underprivileged economic and educational profiles, a community-based study may represent precise results.

## Conclusion

When the research was started, to the best of the authors’ knowledge, assuming that still, no other studies had examined the KAP among university students in Bangladesh concerning monkeypox. Bangladesh’s rapid economic recovery from the COVID-19 pandemic necessitates an immediate understanding of monkeypox’s KAP. It is strongly believed that the findings of this study will provide policymakers with crucial insights for formulating and improvising awareness programs and strategies to manage health emergencies, such as COVID-19 or monkeypox, in the future. This study was carried out to uncover the KAP levels regarding monkeypox among university students in Bangladesh and identify the associated risk factors that determine their levels of KAP. It was found that a significant number of students had moderate to high knowledge and the same levels of attitude and practices regarding monkeypox to protect themselves from the potential risks. There was a significant association between participants’ sex, residence, socioeconomic status, media exposure, and contracting monkeypox. This study recommends adopting a more balanced strategy by increasing the overall knowledge with a cautious attitude and protective and preventive practices regarding monkeypox in rural and urban areas to bolster public awareness regarding the risk of human monkeypox.

## Supporting information

S1 File (DOCX)
