# Supplementary material for: Knowledge, attitude, and practice of university students towards monkeypox in Bangladesh
Source: PLoS One. 2023 Oct 12;18(10):e0287407. doi: 10.1371/journal.pone.0287407 (PMC10569525; doi:10.1371/journal.pone.0287407)
Supplement: S1 File — (DOCX) [file pone.0287407.s001.docx]

**Knowledge, Attitude, and Practice towards Monkeypox among Bangladeshi Students: A Web-based Investigations**

We appreciate your voluntary participation in this survey, which will be beneficial for measuring the knowledge, attitude, and practice toward Monkeypox among Bangladeshi Students. We assure you that your responses will be kept confidential and will not be disclosed.

We, researchers, would like to request you to spend approximately 10 to 15 minutes responding to the e-questionnaire related to Monkeypox. Your participation can assist us with this research, it would be much appreciated. Would you agree to participate in this e-survey and complete the e-questionnaire?

- **Yes**
- **No** (Go to the Submit Button)

**Personal details**

**1. Age (in Year):**

**2. Sex identity:** Male =1, Female = 2, Third sex =3, Prefer not to say=4

**3. Religion:** Islam=1, Hindu (Sanatan) = 2, Other = 3

**4. Years of schooling:** Honors 1^st^ year = 13, Honors 2^nd^ year = 14, Honors 3rd year =15, Honors 4^th^ year / Graduate = 16, Masters = 17, PhD/Post-graduate = 18 and above.

**5. Marital Status:** Never Married = 1, Ever married = 2

**6. Living status:** Alone = 1 With friends = 2, With family = 3

**7. Living location:** Rural= 1, Sub-urban=2, Urban = 3

**8. Division:** Khulna = 1**,** Dhaka = 2**,** Rajshahi= 3, Rangpur = 4**,** Barishal = 5, Chattogram = 6, Mymensingh = 7, Sylhet = 8

**9. How would you rate your overall health status:**  Poor = 1, Fair = 2, Good= 3, Very good =4, Excellent = 5

**10. Have ever been affected by Monkeypox?** Yes =1, No = 2, May be/not sure = 3

**Knowledge information**

| **Sl. No.** | **Statement** | **Response** | | | | |
| --- | --- | --- | --- | --- | --- | --- |
|  |  | **SD**  **(1)** | **D**  **(2)** | **N**  **(3)** | **A**  **(4)** | **SA**  **(5)** |
| 1 | Monkeypox is contagious caused by the monkeypox virus |  |  |  |  |  |
| 2 | *There is a known cure for the virus* |  |  |  |  |  |
| 3 | Homosexuals, bisexuals, and people with multiple sex partners are more at risk |  |  |  |  |  |
| 4 | *Human-to-human transmission occurs only through large respiratory droplets* |  |  |  |  |  |
| 5 | The illness typically lasts for 2–4 weeks |  |  |  |  |  |
| 6 | There is no evidence that COVID-19 vaccinations cause monkeypox |  |  |  |  |  |
| 7 | The first human case of monkeypox was recorded in 1970 in the Democratic Republic of Congo |  |  |  |  |  |
| 8 | The disease symptoms of monkeypox are identical to smallpox |  |  |  |  |  |
| 9 | *The most prevalent symptom of monkeypox is diarrhea* |  |  |  |  |  |
| 10 | The presence of swollen lymph nodes distinguishes monkeypox from smallpox |  |  |  |  |  |

**^Note. SD.^** Strongly Disagree; **^D.^** Disagree; **^N.^** Neutral; **^A.^** Agree; **^SA.^** Strongly Agree

***Attitude Information***

| **Sl. No.** | **Statement** | **Response** | | | | |
| --- | --- | --- | --- | --- | --- | --- |
|  |  | **SD**  **(1)** | **D**  **(2)** | **N**  **(3)** | **A**  **(4)** | **SA**  **(5)** |
| 1 | People should avoid eating undercooked meat or other animal products from infected animals to decrease the potential risk |  |  |  |  |  |
| 2 | Patients with unusual rash, vesicular or pustular lesions, typically along with fever, should alert healthcare providers at the community level |  |  |  |  |  |
| 3 | Infected persons should be isolated from others to reduce the possibility of human-to-human transmission |  |  |  |  |  |
| 4 | Open wounds should be covered, and a mask should be used when in close contact with others |  |  |  |  |  |
| 5 | Infected and uninfected people should avoid touching any wounds that include blisters or pus-filled patches |  |  |  |  |  |
| 6 | People should avoid touching the clothing or bedding of someone with the rash |  |  |  |  |  |
| 7 | Middle-aged people, particularly those with chronic diseases, should be more cautious to avoid getting infected with monkeypox |  |  |  |  |  |
| 8 | The medical staff should be familiar with the symptoms of monkeypox to identify, prevent, and control the upcoming transmission |  |  |  |  |  |

**^Note. SD.^** Strongly Disagree; **^D.^** Disagree; **^N.^** Neutral; **^A.^** Agree; **^SA.^** Strongly Agree

**Practice information**

| **Sl. No.** | **Statement** | **Response** | | | | | |
| --- | --- | --- | --- | --- | --- | --- | --- |
|  |  | **NA**  **(0)** | **N**  **(1)** | **R**  **(2)** | **ST**  **(3)** | **VO**  **(4)** | **A**  **(5)** |
| 1 | I have avoided contact with sick or dead animals with symptoms |  |  |  |  |  |  |
| 2 | I have avoided close contact with anyone who has a rash that includes blisters or pus-filled patches |  |  |  |  |  |  |
| 3 | I wash my hands with soap and water or use an alcohol-based hand sanitizer after coming in contact with an infected individual |  |  |  |  |  |  |
| 4 | I eat thoroughly cooked foods that contain animal meat or any part of it |  |  |  |  |  |  |
| 5 | I wear a face mask when in close contact with someone with symptoms |  |  |  |  |  |  |
| 6 | I always maintain safe sex and monogamy (if applicable) |  |  |  |  |  |  |
| 7 | I avoid sneezing in public spaces and cover my nose when someone sneezes |  |  |  |  |  |  |
| 8 | I don’t share cigarettes (similar to other addictive materials) with others (If applicable) |  |  |  |  |  |  |

**^Note. NA.^** Not applicable; **^N.^** Never; **^R.^** Rarely; **^ST.^** Sometimes; **^VO.^** Very Often; **^A.^** Always

**Media Exposure**

1. **During the past four weeks, how often you were exposed to news and information regarding Monkeypox on electronic and social media - Television, Radio, Facebook, Messenger, WhatsApp, Instagram, Twitter, Skype, Viber, and so on?** Never =1, Occasionally =2, Sometimes =3, Often = 4 , Always = 5
2. **What are the top three (3) MAIN sources of information you have received, regarding Monkeypox?**
3. **How many HOURS do you spend on electronic and social media in a day to get updated** **regarding Monkeypox?**
